# Improving recording and reporting of dementia and frailty via electronic patient record by ambulance staff in a single service (IDEAS)

**DOI:** 10.29045/14784726.2021.12.6.3.31

**Published:** 2021-12-01

**Authors:** Patryk Jadzinski, Helen Pocock, Chloe Lofthouse-Jones, Phil King, Sarah Taylor, Ed England, Julian Cavalier, Carole Fogg

**Affiliations:** University of Portsmouth; South Central Ambulance Service NHS Foundation Trust ORCID iD: https://orcid.org/0000-0002-6752-0807; South Central Ambulance Service NHS Foundation Trust ORCID iD: https://orcid.org/0000-0001-7648-5313; South Central Ambulance Service NHS Foundation Trust ORCID iD: https://orcid.org/0000-0001-8118-3934; South Central Ambulance Service NHS Foundation Trust ORCID iD: https://orcid.org/0000-0001-7736-7183; South Central Ambulance Service NHS Foundation Trust ORCID iD: https://orcid.org/0000-0002-2488-7158; South Central Ambulance Service NHS Foundation Trust ORCID iD: https://orcid.org/0000-0002-8009-2843; Southern Health NHS Foundation Trust; University of Southampton ORCID iD: https://orcid.org/0000-0002-3000-6185

**Keywords:** dementia recording, emergency medical services, electronic patient record analysis

## Abstract

**Background:**

Dementia is common in older adults assessed by ambulance services. However, inconsistent reporting via the patient record may result in this diagnosis being overlooked by healthcare staff further down the care pathway. This can have a deleterious effect on subsequent patient care, increasing morbidity and mortality. We sought to understand how and where ambulance staff would like to record this finding on the electronic patient record (ePR).

**Methods:**

We designed and implemented a survey of ambulance staff in a single service to understand how they identify patients with dementia, how they record dementia on the ePR and how the ePR could be improved to better capture dementia. Scoping questions on frailty were included. The survey was tested using cognitive interviewing. Analysis was conducted using descriptive statistics for closed questions and thematic analysis for open questions as appropriate.

**Results:**

131 surveys were completed; 60% of participants were paramedics and 40% were other grades of front line staff. Participants reported consulting electronic/paper sources, and individuals such as carers involved in the patients’ care, to establish whether dementia had been diagnosed. Frailty assessments were prompted by social context, reduced mobility, a fall or diagnosis of dementia. Staff reported documenting dementia in 20 different areas on the ePR and 46% of participants stated a preference for a designated area to record the information. However, 15% indicated it was not necessary to record dementia or that no ePR changes were required.

**Conclusions:**

We have highlighted the variation in ambulance staff practice in recording of dementia. Alterations to the ePR are required to ensure that dementia is recorded consistently and is easily retrievable. Clearer guidance on when to assess frailty may also enhance information provision to care staff in other sectors, resulting in more appropriate clinical and social care.

## Introduction

Ambulance services provide emergency urgent and unscheduled care in response to 999/111 and healthcare provider calls (South Central Ambulance Service, 2020). Many of these calls are to older people. Due to the complexity of older people’s care, the ambulance service often refers patients to general practitioners and hospital settings, as well as other community healthcare services specific to older people, and adult social care. It is therefore important that information on key diagnoses such as dementia and the presence of frailty are available to all health and social care partners and can be effectively communicated.

Dementia is an increasingly common presentation in older people accessing ambulance services and hospital emergency departments, from pre-diagnosis through to end-of-life scenarios (Buswell et al., 2015; Voss et al., 2018). An audit of the South Central Ambulance Service (SCAS) electronic patient record (ePR) showed that, out of 314,786 ePRs of patients aged 65+ in a 1-year period, 13.5% had ‘dementia’ recorded somewhere in the ePR, increasing to 16.5% of patients aged ≥75 (Pocock et al., 2018). The audit also found that dementia was recorded in 16 different free-text fields, and 38.4% of records had dementia recorded in more than one field. Similar issues were found in another ambulance service, with dementia being recorded across a range of data fields including previous medical history, social or family history and treatment advice or notes (Buswell et al., 2016). This lack of systematic recording may impact on the retrieval of this information by healthcare professionals, reduce the quality of information passed between healthcare services and delay subsequent provision of specialised care.

The SCAS ePR system is a commercial product (Ortivus.com) used by UK ambulance services with local customisation according to needs. It was originally implemented from 2016 across the Trust as part of a national project to move ambulance Trusts away from paper-based systems. The same system is used within the South West Ambulance Service Trust (SWAST) and Northern Ireland. The ePR consists of a hand-held, touchscreen tablet device that uses the Ortivus MMM software to interact with the user via the screen. The screen layout of the MMM software consists of a series of tabs where users can record information using free-text fields, drop-down boxes and pre-configured lists. Currently, the information about dementia can be recorded in a number of locations throughout the record, but there is currently no designated area or specific field on the SCAS ePR where dementia must be recorded.

The question this research sought to answer was ‘How and when do ambulance staff identify dementia and frailty, plus where do they record it and why?’ This information may facilitate improved design of electronic recording systems and associated training.

## Methods

### Study design

The study design was an electronic survey, delivered to a cross-sectional sample of ambulance staff.

### Development and testing of survey tool

The survey tool was developed by the research team with the Patient Public Involvement group and was composed of open free-text and closed-response items. It was piloted with a small group of paramedics familiar with the SCAS ePR, based outside of the study area.

A Think-Aloud Cognitive Interview approach was taken, to understand the users’ perception of the meaning of the questions asked (Beatty & Willis, 2007). Both think-aloud and probing questions were used to understand participants’ interpretation of the questions, a hybrid technique commonly used in the practice of developing and testing questionnaires (Pocock, 2013).

This article adheres to the CROSS survey reporting framework (Sharma et al., 2021).

### Study setting

The study took place in the South East Hampshire division of South Central Ambulance Service between November 2018 and March 2019. SCAS covers the counties of Hampshire, Berkshire, Oxfordshire and Buckinghamshire (approximately 3650 square miles). This represents a combined population of 4.2 million people. In the annual data capture for the year when this study took place, 488,526 calls were made to SCAS. Of these, 80,220 were for incidents located in the South East Division area (SE), with 33,873 of these calls for patients aged ≥65.

### Participants

Emergency front line ambulance ePR users, including nurses, paramedics, student paramedics, ambulance technicians, associate ambulance practitioners (AAPs) and emergency care assistants (ECAs), were invited to participate. All participants recruited were from the South East Hampshire division of SCAS. Purposive sampling was employed to recruit participants during their quarterly team training sessions. These events are part of the staff rota and are compulsory to attend for all team members. Internal and external guest speakers are often invited to these meetings, and they are designed to provide periodic face-to-face updates and training to staff. All teams in the South East node were invited to take part. The invitation was sent by the lead researcher, via email to the Team Leader. One follow-up invitation was sent to teams that had not responded to the invitation, after which no more contact attempts were made. Information about the study was provided via email, two weeks prior to the date of the training session, with a face-to-face presentation at the team session prior to inviting attendees to provide written consent to take part.

### Data collection

Data were collected during team sessions, allowing participants to take as long as required to complete the survey. Study team members were available to assist with technical difficulties or answer questions. Each participant was provided with an electronic tablet. A link to the survey was presented on the tablet which, when clicked, opened a Microsoft Office Form questionnaire (Supplementary 1). Responses were sent to a password-protected Cloud used by SCAS.

### Bias

Selection bias was minimised by inviting all staff of all grades to take part within the study period. By ensuring responses were collected electronically and anonymised, response bias was reduced. The survey was piloted by SCAS paramedics working out of the area where the study was undertaken, in order to minimise instrument bias. The managers of all teams in the South East Hampshire division were approached with an invitation, to offer all teams an equal opportunity to take part in the study.

### Sample size

All teams in the study region were invited to participate. Accounting for absence and leave, the study size was estimated at 100–150 participants.

### Data analysis

Closed questions were analysed with descriptive statistics, using Microsoft Excel. Open-ended questions were analysed with the use of the NVivo Suite (version 12), using principles of thematic content analysis. Data were independently coded and themes identified by two researchers. No a priori themes were postulated, so themes emerged from the data. Differences were settled by a third member of the team.

## Results

### Number and characteristics of participants

**Figure 1:**
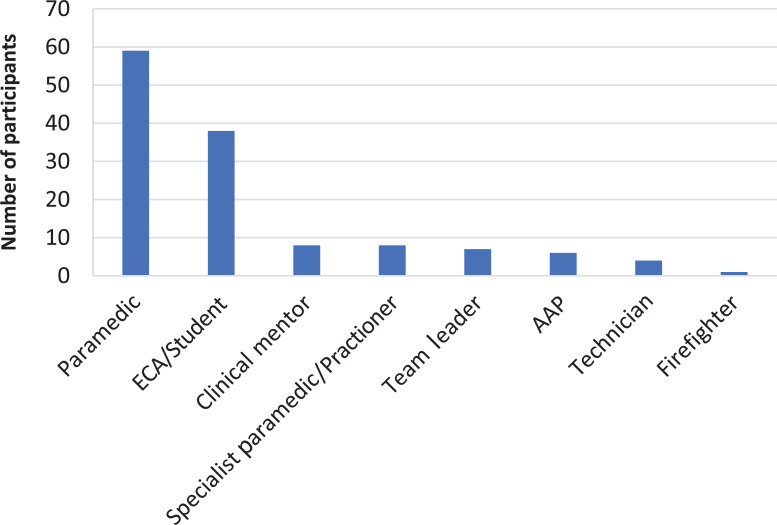
Figure 1. Participant role.

Thirteen teams were approached, of which nine responded and participated in the study. No team declined the invitation and the non-responding teams have offered no explanation for not inviting the research team to their session. 133 individuals were invited to participate and, from this population, 131 (98.5%) participants were recruited, with non-consenting meeting attendees being students who did not feel they had the knowledge to respond. More than half of the participants had worked in the service for more than five years (53.4%, n = 70), 34.4% (n = 45) for one to five years and 12.2% (n = 16) for less than a year. The roles of participants are shown in Figure 1.

### Response to survey questions

#### Recognition or suspicion of dementia

All participants (100%, n = 131) answered an open-ended question aimed at establishing how the staff identify a patient with dementia, and 22 meaningful codes were developed. The dataset was coded accordingly, and two main themes with a further five sub-themes were developed to categorise the data into meaningful clusters (Table 1). The most dominant code for source of information when establishing a history of dementia was history acquired from the family, followed by patients’ paper clinical notes and information from care staff.

**Table 1.** Codes and themes developed to identify how ambulance staff recognise or suspect a patient with dementia.

Theme	Sub-theme	Code	Number of responses relating to code
**History taking**	People	Family	83
		Care staff	44
		Self-disclosure	28
		Neighbours or friends	11
		General practitioner (GP)	9
		Emergency operations centre	2
	Other	Medical history unspecified source	54
		Dementia care home	2
	Paper	Paper clinical notes	42
		Paper social care notes	35
		Current meds	13
	Electronic	Past ePRs	16
		Summary care record	6
**Patient assessment**	Observed characteristic	Confusion	20
		Repetitiveness	11
		Observed characteristic	10
		Memory loss	8
		Cognitive impairment	6
		Inability to recall	1
		Not remembering	1
	Testing the patient	Mental capacity test	5
		Ability to answer questions	4
		Memory test	3

#### Documenting the presence of dementia

The majority of staff (95.4%, n = 125) stated that they would record the presence of dementia on the ePR once this has been identified. Respondents who would not record dementia (3.1 %, n = 4) stated reasons such as not having an appropriate field on the ePR to record it in, feeling that there was no need for it when the patient was being discharged in a care home and, in one case, accidental omission of documentation. Two (1.5%) responses were unrelated to the question and were discarded.

#### Current location of recording dementia on the ePR

Participants were asked on which section of the ePR they currently recorded that a patient has dementia, with the option to record up to three answers in order of preference. Although the ‘Past medical history’ (PMH) (26.7%), ‘Other PMH’ (21.4%) and ‘Neurological’ and ‘Mental health’ (13.7%) tabs on the ePR appear the most commonly selected areas, there was a broad spread of other locations that were also recorded (Table 2).

**Table 2.** The spread and frequency of responses indicating where dementia is currently being recorded on the ePR.

Location name	1st choice	2nd choice	3rd choice	Total
Past medical history	35	7	0	42 (32.1%)*
Other past medical history	28	14	4	46 (35.1%)
Neurological	18	12	1	31 (23.7%)
Mental health	18	9	4	31 (23.7%)
Presenting condition	9	6	4	19 (14.5%)
Examination	6	8	4	18 (13.7%)
Free text	5	12	2	19 (14.5%)
Initial assessment	3	4	4	11 (8.4%)
AMPLE (Allergies, Medications, Past medical history, Last meal, Events preceding)	3	0	0	3 (2.3%)
Unclear location	2	1	1	4 (3.1%)
Disability	1	4	1	6 (4.6%)
Clinical frailty scale	1	1	0	2 (1.5%)
Consent	1	0	0	1 (0.8%)
Impression and plan	0	3	1	4 (3.1%)
Falls risk assessment	0	1	1	2 (1.5%)
Social history	0	1	0	1 (0.8%)
Non-specified free text	0	1	0	1 (0.8%)
Safeguarding referral	0		1	1 (0.8%)
Lifestyle	0	0	1	1 (0.8%)
Total	131	85	29	

* As a proportion of all those who responded (n = 131).

#### Where should dementia be recorded?

When asked to identify where dementia should be recorded, past medical history was identified as the most appropriate location and was preferred by 37.4% (n = 49) of participants (Table 3). This field was followed in order of preference by 14.5% (n = 19) for the medical history field and 11.5% (n = 15) the neurological field. It should be observed that all three answers link to a form of medical history. As regards reasons for the choice of location, 47% (n = 62) of participants felt it was the most appropriate location, 22.1% (n = 29) stated it was to support other healthcare professionals, 16.8% (n = 22) described there being nowhere else to capture this information, 7.6% (n = 10) believed this was part of their medical model, 3.8% (n = 5) referred to the reason for attendance and 2.3% (n = 3) provided an invalid answer that could not be categorised.

**Table 3.** Fields identified by staff where they believe it would be most appropriate to record that the patient has dementia.

Location	Consolidated location	1st choice	2nd choice	3rd choice	Total
Past medical history	Past medical history	49	5	0	54 (41.2%)*
Medical history	Past medical history	19	2	0	21 (16.0%)
Neurological	Neurological	15	3	0	18 (13.7%)
Designated tab	Other designated tab	13	0	0	13 (9.9%)
Mental health	Mental health	8	0	1	9 (6.9%)
Examination	Examination	3	0	0	3 (2.3%)
Patient details front page	Patient information	3	0	0	3 (2.3%)
AMPLE (Allergies, Medications, Past medical history, Last meal, Events preceding)	AMPLE	2	0	0	2 (1.5%)
Capacity – add section	New section	2	0	0	2 (1.5%)
Front page	Patient information	2	0	0	2 (1.5%)
Nil answer	Not answered	2	0	0	2 (1.5%)
Presenting condition	Presenting condition	2	0	0	2 (1.5%)
Primary assessment	Primary survey	2	0	0	2 (1.5%)
Free text	Other designated tab	1	4	2	7 (5.3%)
Further notes under Glasgow Coma Scale (GCS) score	Vital signs	1	1	0	2 (1.5%)
Complex question	Other designated tab	1	0	0	1 (0.8%)
Conditions	Presenting condition	1	0	0	1 (0.8%)
Frailty	Frailty	1	0	0	1 (0.8%)
Observations	Vital signs	1	0	0	1 (0.8%)
Presenting Condition (PC), History of Presenting Condition (HPC), PMH, Mental health tab	Presenting condition	1	0	0	1 (0.8%)
Social history	Patient information	1	0	0	1 (0.8%)
Tick box for learning difficulties	Patient information	1	0	0	1 (0.8%)
Separate dementia tab	New section	0	28	3	31 (23.7%)
Mental health history	Mental health	0	8	0	8 (6.1%)
Separate tab near frailty score	New section	0	2	0	2 (1.5%)
Cognitive tab	New section	0	1	0	1 (0.8%)
Highlighted	New section	0	1	0	1 (0.8%)
Specific adult Mental Health (MH) tab	Mental health	0	1	0	1 (0.8%)
Specific yes/no tab	New section	0	1	0	1 (0.8%)
Subheading with tab	New section	0	1	0	1 (0.8%)
Yes/no tab	New section	0	1	0	1 (0.8%)
Consent	Patient information	0	0	1	
					1 (0.8%)
Total		131	59	7	

* As a proportion of all those who responded (n = 131).

#### What would make it easier to record dementia on the ePR?

**Figure 2:**
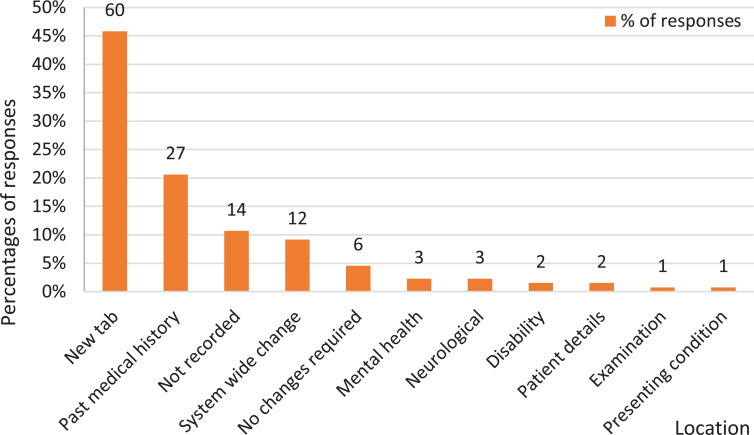
Figure 2. Responses indicating what would make it easier to record dementia on the ePR.

When asked what would make it easier to record dementia on the ePR, 45.8% (n = 60) of participants asked for a separate, designated tab, which was the dominant answer (Figure 2). Although other suggestions were listed, an overwhelming majority was associated with the presence of a designated button, tab or tick box, allowing the operator to explicitly record that a patient has dementia.

#### Assessment of frailty

Seven themes were identified in response to the question ‘In what circumstances would you assess for frailty?’ (Table 4). The most prevalent theme indicated by 47.3% (n = 62) of responses was associated with advanced age.

**Table 4.** Themes and supporting codes of when staff would consider assessing frailty.

	Code	Total count	% of respondents
**1**	Reduced mobility	23	17.6
**2**	Social situation Social/personal challenges Care needs increased: *Recent decline* *Increasing dependence* Lives alone Living conditions Concern for welfare: *Patient/relative concerns*	37	28.2
**3**	Disposition Discharged on scene GP referral Hospital admissions Safeguarding/falls	16	12.2
**4**	Previous Medical History (PMH) Dementia	17	13
**5**	Elderly	62	47.3
**6**	Physical situation Clinically relevant Confused Post injury Unable to converse Visibly frail	14	10.7
**7**	Type of incident Falls: *Unexplained fall* *Frequent fall* End-of-life care criteria Generally unwell Medical emergency	30	23

## Discussion

We found that ambulance staff report recognising or suspecting that a patient has dementia while taking a history ‘on scene’, with family members and care staff being the most frequent sources of information. Hard copies of clinical notes and social care notes found on scene were the next most accessed source of information. Fewer respondents acknowledged the role of self-disclosure by the person with dementia, and only 16/131 (12.2%) respondents indicated that they would use ePRs from previous ambulance service attendances as a source of information. This is consistent with the previous work of Voss et al. (2018), who recognised the importance of ‘on scene’ information sources to ambulance crews rather than reliance on wider healthcare records.

Although electronic patient records are designed to streamline the process of recording and sharing data and to enhance patient care and safety, evidently they do not always produce the intended outcomes, as users’ perception of the suitable location of recording dementia in this particular study shows broad disparity. Ambulance staff are required to be information analysts, having to make sense of each scene to which they are called. Whether they suspect dementia and look for evidence to support/refute hypotheses (top-down approach) or piece together the information they find to arrive at conclusions (bottom-up), our data showed that multiple information sources are used to support their recording of dementia. This is intrinsic to the sense-making process developed by Pirolli and Card (2005). The adopted version of this model is illustrated in Supplementary 2. A user-friendly ePR should act as both a recording framework and a prompt tool. Certain findings will act as prompts to seek other related information that may or may not fit the schema of dementia. Therefore, using software that allows for multiple locations for recording dementia may lead to missed prompts for further probing which can contribute to vital information being missed by staff. This, in turn, could have a detrimental impact on patient safety since patients with dementia are likely to have negative outcomes when admitted to hospital and are at further risk of deterioration if their specific needs are not addressed (Fogg et al., 2018). All individuals involved in the care of dementia patients must, therefore, be aware of the diagnosis of this condition in order to better address this vulnerable group’s complex needs. Frailty assessments in emergency departments and hospital wards are becoming more common due to the increased risk of poor hospital outcomes of people with frailty, but there are challenges to completing assessments in a timely way (NIHR Dissemination Centre, 2017). It is possible that an assessment in pre-hospital care may provide at least a guide to emergency department or admitting ward staff to provide adequate care during the initial hours of admission.

Frailty assessment is an optional section on the ePR system used by participants, with advanced age being the factor most likely to prompt assessment and recording of frailty. A history of dementia prompted an assessment of frailty in few cases, suggesting ambulance staff may perceive age as a greater risk factor than dementia for frailty. It may be that the prominence of a patient’s age, on the opening tab of a patient record, and the designated section for frailty act as visible prompts for consideration of frailty. Recognition of frailty can provide useful information to clinicians when considering a patient’s risks and resilience as part of a holistic assessment of their needs. If a designated area for dementia recording were placed next to the frailty section in the ePR, this could increase the likelihood of both sections being completed, where clinically indicated.

Our study found that most respondents would record the presence or suspicion of dementia; yet, with the absence of a dedicated section for dementia on the ePR, it is recorded across 20 different ePR sections, which closely correlates with the findings of Pocock et al. (2018). This inconsistent location of recording suggests that the current system, the ePR, is suboptimal, as information regarding a patient’s dementia may not be readily apparent. This was clearly recognised by staff in our survey, the majority of whom preferred a single place to record dementia diagnosis. Inconsistent recording represents a risk if receiving medical staff cannot reliably source this information following clinical handover, and may contribute to the significant problem of under-coding of dementia during hospitalisation among most Organisation for Economic Co-operation and Development (OECD) countries (OECD, 2018). A recent retrospective review of medical records over a 10-year period found that among patients known to have dementia, its recognition in subsequent hospitalisation was influenced by the reason for admission (Cappetta et al., 2020). Patients were more likely to have their dementia documented when they were admitted to hospital for falls and less likely for medical conditions including pneumonia and urinary tract infection (UTI). Furthermore, their study also reported that the over 65s were more likely to be admitted to the emergency department by ambulance, and patients presenting with delirium were 20% more likely to have dementia actively managed. Ensuring that information on a dementia diagnosis can be found in a consistent location on the ePR for any patient admitted by ambulance, regardless of their presenting complaint, may prevent a delay in awareness, and subsequent appropriate management, of this complex progressive condition.

### Strengths and limitations

The use of individual tablet devices to capture participants’ responses provided a secure method of data capture and transfer. This enabled the researchers to capture the individual responses of each group of participants, ensuring that every voice was heard and given equal weight.

A potential weakness of the study was that staff were recruited from one geographic area and their experiences may not be entirely the same as staff in other areas in the Trust. However, all staff across the Trust receive the same role-specific statutory and mandatory training and use the same ePR system regardless of their location, so the results are arguably transferable within this Trust, and might be applicable to other services which use the same system.

Closed-response options may have resulted in participants not being able to find an answer that reflected their true opinion. We balanced this by also including free-text response options so that we did not limit or influence participants’ suggestions about how dementia and frailty should be captured.

Recommendations for improvements to the ePR were generated by users themselves in this study. The importance of adopting user-centred interactive design has previously been highlighted (Horsky et al., 2012), as has the need to understand how well the existing model works before making changes (Jafar et al., 2018). Our study is an early attempt to engage in this process and offer findings that could aid the development of how ambulance services record dementia in the future, using the ePRs. However, it should be acknowledged that this study represents regional findings that may not be generalisable to all ambulance services.

## Conclusions

Based on the findings of our study, we recommend implementation of a designated area on the ePR to record dementia and frailty, as all care providers involved in the patient’s journey could refer to and record the information in the same place, thus minimising the risk of vital information being missed. This may also prompt increases in frailty assessments. To inform the broader community, we recommend a larger scale study of this design to be carried out across multiple organisations, in order to validate our findings or offer novel contributions to the evidence base. Further evaluation of the ePR after implementation of designated areas for recording dementia and frailty, and follow-up studies with healthcare professionals, families and patients as to the impact of collecting and transferring the information, are essential.

## Author contributions

All authors were involved in the design of this study. PK extracted the data and designed the online questionnaire, and PK, CF, PJ, HP, CLJ and ST performed data analysis. All authors were involved in the interpretation of data, and in the writing of the manuscript. All authors have approved the final manuscript. PJ acts as the guarantor for this article.

## Conflict of interest

None declared.

## Ethics

This study has been approved by the Health Research Authority, IRAS project number 249651, and has also received favourable opinion from SCAS’s Clinical Review Group. Although the participants were offered the opportunity to participate in a draw for a chance to win one of five £20 Amazon vouchers, this amount and type of reward were not deemed significant enough to entice participation solely for materialistic gains. This was a token to express gratitude for the time taken by participants, which is frequently practised in the field of research.

## Funding

This study has been awarded a small research grant from the College of Paramedics (CoP). The CoP has had no further association with or input into this study, and consequently no conflict of interest can be inferred from this organisation offering financial support for this project.

## References

[bibr_1] Beatty P. C. & Willis G. B. (2007). Research synthesis: The practice of cognitive interviewing. *Public Opinion Quarterly*, 71(2), 287–311.

[bibr_2] Buswell M. Amado S. Goodman C. William J., Fleming J., Lumbard P. & Prothero L. (2015). Does dementia matter: Is dementia an important factor in 999 call-outs to older people? *Emergency Medicine Journal*, 32(6), P007.

[bibr_3] Buswell M. Lumbard P. Fleming J. Ayres D. Brayne C. & Goodman C. (2016). Using ambulance service PCRs to understand 999 call-outs to older people with dementia. *Journal of Paramedic Practice*, 8(5), 246–251.

[bibr_4] Cappetta K. Lago L. Potter J. & Phillipson L. (2020) Under-coding of dementia and other conditions indicates scope for improved patient management: A longitudinal retrospective study of dementia patients in Australia. *Health Information Management*. Epub ahead of print, 23 January. https://doi.org/10.1177/1833358319897928. 10.1177/1833358319897928 31971019

[bibr_5] Fogg C. Griffiths P. Meredith P. & Bridges J. (2018). Hospital outcomes of older people with cognitive impairment: An integrative review. *International Journal of Geriatric Psychiatry*, 33(9), 1177–1197. 29947150 10.1002/gps.4919 PMC6099229

[bibr_6] Horsky J. Schiff G. D. Johnston D. Mercincavage L. Bell D. & Middleton B. (2012). Interface design principles for usable decision support: A target review of best practices for clinical prescribing interventions. *Journal of Biomedical Informatics*, 45(6), 1202–1216. 22995208 10.1016/j.jbi.2012.09.002

[bibr_7] Jafar A. J. N. Fletcher R. J. Lecky F. & Redmond A. D. (2018). A pilot of a UK Emergency Medical Team (EMT) medical record during a deployment training course. *Prehospital and Disaster Medicine*, 33(4), 441–447. 29962356 10.1017/S1049023X18000468

[bibr_8] NIHR Dissemination Centre. (2017). *Themed review. Comprehensive care. Older people living with frailty in hospitals*. https://evidence.nihr.ac.uk/themedreview/comprehensive-care-older-people-with-frailty-in-hospital/.

[bibr_9] Organisation for Economic Co-operation and Development (OECD). (2018). *Care needed: Improving the lives of people with dementia*. OECD Health Policy Studies. OECD Publishing.

[bibr_10] Pirolli P. & Card S. (2005). *The sensemaking process and leverage points for analyst technology as identified through cognitive task analysis*. https://www.e-education.psu.edu/geog885/sites/www.e-education.psu.edu.geog885/files/geog885q/file/Lesson_02/Sense_Making_206_Camera_Ready_Paper.

[bibr_11] Pocock H. (2013). Adaptation of a tool measuring attitudes towards pain in paramedics. *International Emergency Nursing*, 21 210–215. 23830373 10.1016/j.ienj.2012.07.003

[bibr_12] Pocock H. Jadzinski P. Taylor-Jones C. King P. England E. & Fogg C. (2018). A clinical audit of the electronic data capture of dementia in ambulance service patient records. *British Paramedic Journal*, 2(4), 10–18. 33328796 10.29045/14784726.2018.03.2.4.10 PMC7706762

[bibr_13] Sharma A. Minh Duc N. T. Luu Lam Thang T. Nam N. H. Ng S. J. Abbas K. S. Huy N. T., Marušić, A., Paul C. L. Kwok J. Karbwang J. de Waure C. Drummond F. J. Kizawa Y. Taal E. Vermeulen J. Lee G. H. M. Gyedu A. To K. G., … Karamouzian M. A. (2021). Consensus-based Checklist for Reporting of Survey Studies (CROSS). *Journal of General Internal Medicine*. Epub ahead of print, 22 April. https://doi.org/10.1007/s11606-021-06737-1. 10.1007/s11606-021-06737-1 PMC8481359 33886027

[bibr_14] South Central Ambulance Service. (2020). *999*. https://www.scas.nhs.uk/our-services/999-emergency/.

[bibr_15] Voss S. Brandling J. Taylor H. Black S. Buswell M. Cheston R. Cullum S. Foster T. Kirby K. Prothero L. Purdy S. Solway C. & Benger J. R. (2018). How do people with dementia use the ambulance service? A retrospective study in England: The HOMEWARD project. *BMJ Open*, 8(7), 1–8. 10.1136/bmjopen-2018-022549 PMC6074617 30068624

